# The Use of Advanced Glycation End-Product Measurements to Predict Post-Operative Complications After Cardiac Surgery

**DOI:** 10.3390/jcm14176176

**Published:** 2025-09-01

**Authors:** Divya S. Agrawal, Jose C. Motta, Jason M. Ali

**Affiliations:** 1 School of Clinical Medicine, University of Cambridge, Cambridge CB2 0SP, UK; dsa32@cam.ac.uk; 2 Department of Cardiothoracic Surgery, Royal Papworth Hospital NHS Foundation Trust, Papworth Road, Trumpington, Cambridge CB2 0AY, UK; jose.motta@nhs.net

**Keywords:** advanced glycation end-products, post-operative complications, morbidity, risk, prognosis, cardiac surgery

## Abstract

**Background/Objectives**: Frailty is increasingly recognised as an important contributor to outcomes following cardiac surgery. There are various measures of frailty described, but many include subjective assessments impacting reliability and reproducibility of measurement. A potential biomarker: advanced glycation end products (AGEs) have been suggested to closely correlate with frailty. This may offer the opportunity to objectively measure frailty and have potential use in preoperative risk assessment. The objective and aim of this narrative review is to assess the association between AGEs and outcomes following surgery, in order to evaluate the use of AGEs for preoperative risk assessment. **Methods**: This review involved searching five databases including the following: MEDLINE (through Ovid), Embase, Cochrane, ClinicalTrials.gov, and a specified Google Scholar search for studies published between database inception and 20 February 2025. The 1142 identified articles were then subjected to various inclusion and exclusion criteria. This exclusion criteria included all articles that were not in the English language, studies involving patients under 18 years of age, and studies that were incomplete or for whom the data was not yet available. This left 11 articles for which a ‘related articles’ search was performed on Google Scholar on 6 March 2025, as per the PRISMA-S extension guidelines, to obtain all relevant articles available. In the end, data analysis was conducted on 13 articles with a total of 2402 participants. These were categorised by type of surgery before analysis was performed for each surgical category. The quality of evidence was assessed using ROBINS-I tool and a risk of bias table has been provided. This study was provided no external sources of funding. **Results**: Four out of the five studies in cardiac surgery showed a statistically significant association between AGE levels and post-operative complications and outcomes. This association was also seen across thoracic and general surgery. Association was demonstrated with various post-operative complications as well as mortality. These relationships are supported by various pathophysiological mechanisms, including the ability of AGEs to induce oxidative stress, activate inflammatory mediators, and cause endothelial dysfunction. **Conclusions**: There is a body of evidence supporting the association between AGEs level and cardiac surgical outcomes. This objective measure of frailty could have significant utility in preoperative risk assessment and offer the opportunity to identify patients who will benefit from undergoing prehabilitation.

## 1. Introduction

Frailty is an increasingly recognised entity which is seen to impact the outcomes following cardiac surgery, with frail patients being found to suffer greater morbidity and mortality [1]. There are various measures of frailty, with many being rather subjective, which can limit their utility in accurately and reproducibly categorising the degree of frailty. To improve the reliability of frailty assessment, biomarkers have been sought which can be used as a surrogate for frailty and potentially predict outcomes following surgery. One such biomarker which has gained popularity is the measurement of ‘advanced glycation end products’ (AGEs). AGEs are heterogenous molecules that are formed through non-enzymatic glycation reactions (Maillard reactions) of biological macromolecules like lipids, proteins, and nucleic acids [2]. AGEs have been implicated in the pathophysiology of disease through a diverse range of mechanisms, including the following:

(A) Non-receptor-mediated interactions with lipoproteins or the extracellular matrix (ECM) can cause direct disruption of structural stability of tissues by forming cross-links [3]. This includes interactions with blood vessel walls which can increase vascular permeability and lead to the formation of reactive oxygen species (ROS) [4].
(B) Receptor-mediated binding and activation of ‘receptors of advanced glycation end products’ (RAGE). This leads to oxidative and inflammatory cascades being triggered, stimulating the production of cytokines like tumour necrosis factor alpha (TNF-alpha) and various interleukins [5], thereby enabling the development of inflammatory disorders.

Through such mechanisms, AGEs have been implicated in a wide range of diseases including diabetes mellitus [6], various forms of cancer [7,8,9], and various cardiovascular diseases [10].

Currently, there are two predominant methods in use to measure AGE levels: (i) measuring the levels of the receptors of advanced glycation end products (RAGEs) using enzyme-linked immunosorbent assay (ELISA), or (ii) measuring AGEs using skin autofluorescence (sAF). The latter technique of sAF relies on the use of a skin AGE reader. AGEs in the skin interact with UV (ultraviolet) light that causes the light to be reflected and emitted, and it is this autofluorescence that can be detected by the skin reader to calculate AGE levels [11]. Several studies corroborate the ability of sAF measurements to estimate the AGE levels by showing that sAF positively correlates with levels of AGEs measured through skin biopsies or through measurements from immunohistochemistry of arteries and nerves [12,13].

## 2. AGEs and Frailty

There is accumulating evidence that the level of AGEs is associated with frailty and, therefore, can be used as a means of frailty assessment.

There are multiple methods described to quantify frailty. Amongst these, two of the most common methods use a multi-factorial approach to appraise frailty. The first is Fried’s frailty phenotype, that considers frailty to be defined as having three or more of five different pre-defined components of weak grip strength, slow gait speed, low physical activity level, self-reported exhaustion, and low weight, to assess physical health [14]. Alternatively, Rockwood’s Frailty Index focuses on defining frailty through an accumulation of deficits, including both physical and cognitive [15].

Table 1 highlights that studies have shown that skin autofluorescence (sAF), as a measure of AGEs, is associated with frailty as defined by both Fried’s criteria and Rockwood’s concept.

Table 1 shows that associations between sAF and frailty have been seen, even after adjusting for potential confounding variables, such as age and socioeconomic status. The studies highlight association with both lower physical functioning and a decline in cognitive ability. There is evidence to suggest that AGEs may be more strongly associated with certain components of frailty than others. One study found that amongst the five components of frailty, as defined by Fried, slowness of walking speed and unintentional weight loss were associated with AGEs [16]. Furthermore, studies suggest that the association between AGEs and frailty may be stronger in certain patient groups. For example, one study found that the association between sAF and both cognitive impairment and decline in physical functioning was higher in men than women [27].

What appears clear, though, is that AGEs do seem to be correlated with frailty. There are several proposed mechanisms. The correlation between AGEs and cognitive decline may be explained by their accumulation in the brain as patients age [28] or by the fact that AGEs have been implicated in neurofibrillary tangles [29] that can be found in conditions with cognitive impairment like Alzheimer’s. The relationship between AGEs and physical function decline may be explained by the ability of AGEs to cross-link to collagen in bones and elastin in skin [3], which may deteriorate their remodelling ability and quality.

Given the apparent association between AGEs and frailty, and the association between frailty and outcomes following surgery, the aim of this review is to investigate the association between AGEs and outcomes following surgery to assess the utility of this more objective assessment of frailty as a means of predicting the likelihood of post-operative morbidity and mortality following cardiac surgery.

Objective: To evaluate the utility of AGEs for preoperative risk assessment by synthesising evidence on the association between the levels of AGEs and post-operative complications, across different surgeries, in all adult patients above 18 years of age.

## 3. Materials and Methods

A review of the published literature was performed using PRISMA 2020 guidelines based on the PICO (population, intervention, comparison, and outcomes) framework shown in File S1 (provided in the Supplementary Materials).

### 3.1. Search Strategy

Potential studies for inclusion were identified from 6 sources, using a combination of electronic databases, clinical trials, and online sources. The databases searched were MEDLINE (through Ovid), Embase, Cochrane, and Scopus. The study registry ClinicalTrials.gov was used to identify any ongoing or unpublished trials. And a Google Scholar search was used to further supplement the findings and to reduce bias from only including publications with positive results. All these search strategies were performed on the 20 February 2025.

**Figure A1:**
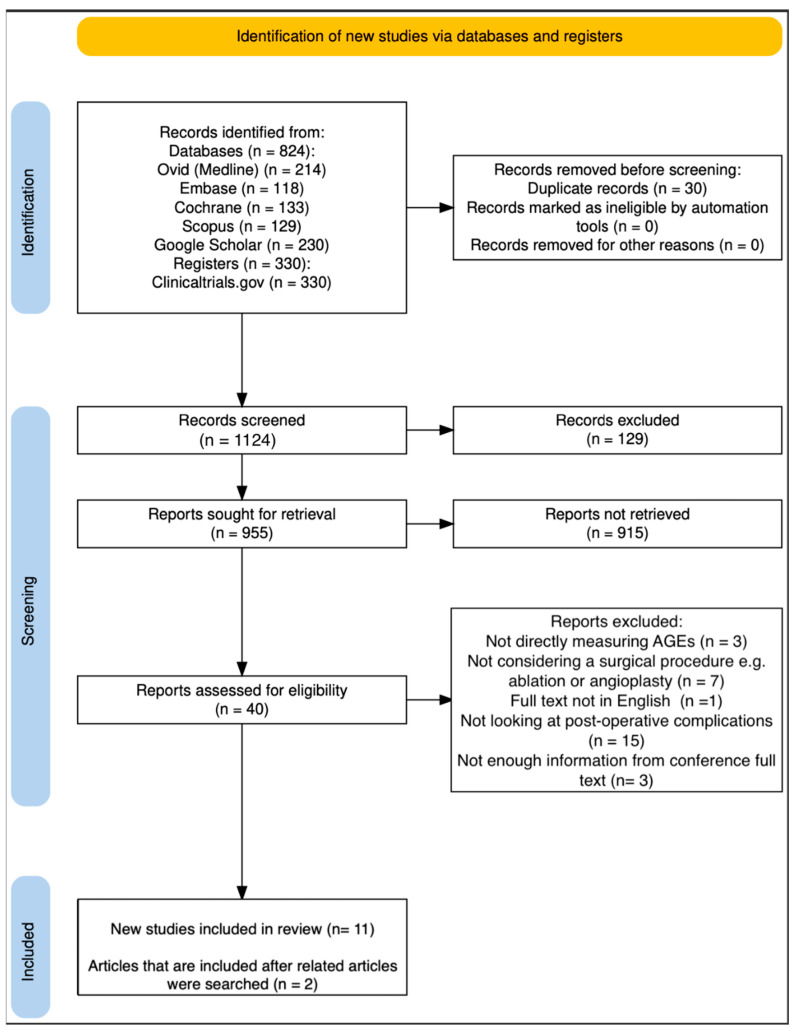
PRISMA Flowchart of Search and Selection Process.

The search strategy for each source or database is fully detailed in File S2 (in the Supplementary Materials) as is the search strategy development process. A PRISMA flowchart detailing the results of the search and selection process are depicted in Figure A1 (provided in the Appendix A).

### 3.2. Study Selection

After de-duplication, 1124 articles remained. The articles were then selected via a two-step process using two reviewers, who worked independently. First, all studies were screened based on the relevance of their abstracts and titles. Any articles that were not related to both AGEs and post-operative complications were excluded. This left 40 articles in all, from which a full text analysis was conducted and, once again, reviewed by two reviewers independently to increase reliability. This full text analysis also removed any articles that were not deemed relevant to both AGEs and post-operative complications. Throughout the process, in the event of disagreements, consensus was reached by discussion and consultation of the third researcher. For the 11 papers that then remained, a ‘related articles’ search was performed on Google Scholar on the 6 March 2025, as per the PRISMA-S extension guidelines, to screen for secondary sources of data. This was an important step since AGEs is a relatively new concept and the literature on the subject is limited, and performing this ensured that all possible studies that were relevant to our narrative review could be found.

Conference papers were included if they were relevant, if they had information about the methodology (e.g., how AGEs were measured, control variables used), specified surgery type, and if results were available. This left 13 articles in total, from which data was subsequently extracted. Table 2 summarises the information that was extracted from these 13 studies and Table S1 (provided in the Supplementary Materials) provides the summary statistics in greater detail.

For completion, Table 3 summarises the studies that, upon initial inspection, appeared to meet the inclusion criteria but were then excluded. The reasoning behind the exclusion is also elucidated.

### 3.3. Data Extraction

For each of the 13 articles, information pertaining to the methodology of the papers was extracted—including the following: sample size, country of study, how AGEs were measured, when AGEs were measured, and what post-operative complications and outcomes were measured and within what time frame; what confounding variables were considered. Furthermore, the association or statistical relationship between the value of AGEs and post-operative complications was also recorded. Note that the outcomes recorded included all the post-operative complications that the study reported, as explained by the PICO framework in File S1. In studies where multiple post-operative outcomes were mentioned, all such outcomes were recorded. Additionally, if there were multiple multivariate models provided to analyse the post-operative complication, then the model considering the greatest number of confounding factors, i.e., the most fully adjusted model, was analysed. This data extraction also involved two reviewers and the results were compared, with any discrepancies being resolved through discussion once again.

### 3.4. Quality Appraisal of Included Articles

To assess the risk of bias in the included studies, the ROBINS-I framework [53] was used since these studies were all either prospective or retrospective cohort studies, with the exception of the study by Choi et al. [37] that was a randomised control trial. The ROBINS-I framework addresses 7 different domains including the following: bias due to confounding factors, bias in selection of participants into the study, bias in classification of interventions, bias due to deviations from intended interventions, bias due to missing data, bias in measurements of outcomes, and bias in selection of the reported result [53]. Two researchers independently applied this tool to the 13 articles. If there were any differences in the judgement of the risk of bias, then discussion was used to reach an agreement. A summary of this risk of bias appraisal is provided in Table S2 (provided in the Supplementary Materials) alongside explanations of why a specific classification was given. The overall risk of bias was determined by taking the highest risk of bias in any of the assessed domains. It was difficult to assess any reporting biases. This was because AGEs is a relatively new area in the field, and so there is little research in the field. Because of this limited number of studies, it was inappropriate to conduct Egger’s regression or Begg’s rank correlation test [54]. And since the effect measures of the studies were very varied, and conversion of effect measures would involve too many assumptions, a funnel plot was not deemed informative either [55]. However, we assessed the outcome reporting biases. To assess the outcome reporting bias, we compared the outcomes that were reported in the methods section of the report to the results section.

In addition to this, two reviewers independently assessed the certainty of evidence using the GRADE framework [54]. In the event of a discrepancy between the judgement of certainty, discussion was used to reach consensus. The certainty of evidence was rated as either high, moderate, low, or very low. Since all the included articles were either retrospective or prospective cohort studies, the studies were allocated a low confidence level as per the GRADE framework [54], with the exception of the study by Choi et al. [37] since this was a randomised controlled trial and so was given a high certainty to begin with instead. The studies’ certainty rating was upgraded based on the following criteria: large magnitude of effect, dose response gradient, or direction of plausible bias. Additionally, the studies’ certainty was downgraded based on the following criteria: risk of bias, inconsistency, or indirectness. The studies’ certainty assessment alongside the explanation for this assessment are provided in Table S3 (provided in the Supplementary Materials).

## 4. Results

### 4.1. Main Study Characteristics Assessment

The findings from the 13 articles are summarised in Table 2. There were five studies investigating association between AGEs and outcomes following cardiac surgery [30,31,32,33,34]. To further understand the association between AGEs and post-operative outcomes more generally, we have also included analysis of papers examining this in other surgical specialities. To this end, we report on five studies exploring outcomes in general surgery [35,36,37,38,39] and three studies that explored outcomes following thoracic surgery [40,41,42].

Amongst these studies, seven used ELISA to estimate AGE levels [30,31,36,37,40,41,42], with all using serum or plasma RAGE levels for the ELISA, with the exception of Simm et al. whose study measured the levels of a particular AGE: carboxymethylysine within pericardial fluid [30]. Reichert et al. used both sAF and ELISA methods to measure the AGE levels [32], whilst the remaining five studies used a skin autofluorescence reader alone [32,34,35,38,39].

A range of post-operative complications and outcomes were measured within these 13 papers. These included the following: intensive care unit admission, hospital length of stay (LOS), cardiac complications like new atrial fibrillation or myocardial infarction, renal complications like acute kidney injury, incisional hernias, transplant graft loss, mortality, reoperation rates, respiratory insufficiency, duration of mechanical ventilation, development of bronchiolitis obliterans syndrome (BOS), and acute exacerbation of fibrotic lung disease (AE-ILD) [30,31,32,33,34,35,36,37,38,39,40,41,42].

The studies used a range of different effect measures to assess the association between AGEs and post-operative complications. Most studies used just one type of effect measure to assess this relationship; however, some used multiple. Spearman’s rank correlation coefficient was used by seven studies [30,31,32,35,36,37,40]; odds ratio was used by one study [38]; mean differences was used as the effect measure by six studies [31,33,35,39,41,42]; and one study used risk ratios [34]. Since these effect measures utilised a variety of different variables including binary, continuous, and qualitative data, too many assumptions would have to be made to convert them to one standardised effect measure. For this reason, no such conversion was made, and it was deemed inappropriate to conduct a meta-analysis of the data. In the future, when more data is collected on AGEs and post-operative outcomes, this could prove an invaluable area of research. Additionally, there was a consensus amongst the researchers for a significance level of 0.05 to be used, since a higher *p*-value would increase the risk of a type I error being made and a lower *p*-value would increase the risk of a type II error, and so α = 0.05 was deemed most optimal.

In order to evaluate and best compare the association between AGEs and post-operative complications, we came to a consensus of categorising and comparing studies that involved similar types of surgery (i.e., surgeries operating on the same types of organs or organ systems), to remove this potential confounding factor. The analysis below, therefore, analyses each type of surgery separately. Since the effect measures, sample sizes, confounding variables, and control variables amongst these studies were highly variable, the study data was not amenable to the use of alternative statistical synthesis methods.

#### 4.1.1. AGEs as Predictors of Post-Operative Complications in Cardiac Surgeries

There were five studies in cardiac surgery. Simm et al. found that there was a statistically significant, direct correlation between AGEs and ventilation time in the oldest tertile of patients [30]. The paper also found that AGEs were associated with an increased level of cardiac complications (*p* = 0.018), again, in the oldest tertile of patients; however, a statistical significance was not found with pulmonary complications [30], suggesting that AGEs may have better prognostic value for certain types of complications. Interestingly, Creagh Brown et al. found that although preoperative levels of sRAGE remained the only variable with an independent relationship with prolonged hospital stay and LOS, no such correlations were found with post-operative sRAGE levels [31]. This may suggest that when the measurement of sRAGEs is done, in relation to the surgery, may also affect their prognostic value for clinicians. Hofmann et al. found that AGEs were associated with higher rates of morbidity, even after age and lower left ventricular ejection fraction were considered [32]. The study also reported that sAF could independently predict in-hospital mortality [32]. Reichert et al. found that both sAF and sRAGE levels were associated with poorer cardiovascular outcomes and an increased risk of new adverse cardiovascular and cerebrovascular events on univariate analysis, but they were not found to be independent predictors on multivariate analysis [33]. Smoor et al. found that sAF was an independent predictor of both death and disability one year after cardiac surgery [34]. Additionally, higher sAF or AGEs were associated with higher levels of dependent living and aspects of frailty as well highlighting the association between AGEs and frailty [34]. From these studies, there appears to be strong evidence of an association between AGEs and post-operative complications following cardiac surgery, with four out of the five studies demonstrating an independent association on multivariate analysis. The final study did not demonstrate AGEs to be significant on multivariate analysis but showed significant results on univariate analyses.

#### 4.1.2. AGEs as Predictors of Post-Operative Complications in General Surgery

There were five studies exploring AGEs in patients undergoing general surgery [35,36,37,38,39]. Pol et al. found that there was an association between preoperative sAF measurements and post-operative complications measured up to 30 days after surgery [35]. Interestingly, perioperative increases in sAF were also associated with post-operative complications, with higher increases in sAF being associated with a greater number of complications [35]. This would suggest that not only are the absolute values of AGEs useful predictors of complications, but there may be prognostic value in monitoring their rate of change as well. Neto et al. did not find an association between sRAGE levels and post-operative pulmonary complications [36]. However, this study only focused on pulmonary complications and did not factor in other types of complications. Choi et al. found that both intra- and post-operative measurements of sRAGE could be used to predict post-operative respiratory complications in patients undergoing laparoscopic colectomy [37]. Additionally, Krasnodebski et al. found that sAF could independently predict AKIs after liver resection, even after confounding variables were considered [38]. Morawski et al. found that there was insufficient evidence to suggest a correlation between sAF and the specific post-operative complication of incisional hernias [39]. It is important to note that this paper only explored this longer-term complication as opposed to shorter term post-operative complications that other papers considered. Overall, in studies involving general surgery, three of the studies demonstrated statistical significance between AGEs and post-operative outcomes, and the two papers that did not were focused on specific outcomes: one being centred on incisional hernias and the other focusing on only pulmonary complications.

#### 4.1.3. AGEs as Predictors of Post-Operative Complications in Thoracic Surgeries

There were three studies exploring AGEs in thoracic surgery. All three found that there was statistical significance between AGEs and different post-operative complications and outcomes [40,41,42]. Shah et al. found that sRAGE levels measured at 24 h after lung transplantation were significantly associated with the time to develop bronchiolitis obliterans syndrome (BOS), even after factoring in confounders [41]. In contrast, Calfee et al. found no significant relationship between AGEs and the development of BOS or mortality 1 year after surgery [40]. However, they found that AGE levels were significantly correlated with the duration of mechanical ventilation and length of ICU stay, with the latter increasing by 1.8 days, on average, as the RAGE levels doubled [40]. Nakao et al. also found that sRAGE levels were significantly associated with higher rates of post-operative AE-ILD, after adjustments for confounders [42]. However, interestingly, this was the only paper where lower levels of sRAGE were correlated with higher rates of post-operative outcomes, whereas all other studies showed higher levels of AGEs increasing risk of complications. This paradoxical finding might be explained by the fact that this study measured the levels of sRAGE an entire month after the operation whereas most of the other papers measured levels of AGEs within 24–48 h of the surgery. Overall, all thoracic surgery studies showed statistical significance between the AGEs and different post-operative complications, with one paper showing a paradoxical relationship which might be explained by when the sRAGE measurements were made relative to the surgery.

## 5. Discussion

Studies exploring AGEs and post-operative outcomes demonstrate that there is a strong correlation between AGEs levels and the incidence of post-operative outcomes across a range of surgical specialties, including cardiac surgery, suggesting that this may be a useful tool to assess patients prior to surgery to help with risk assessment. Mortality risk assessment is well established in cardiac surgery with EuroSCORE [56] and the STS (Society of Thoracic Surgeons) scores [57], with both being widely used and validated models for assessing the risk of mortality. However, cardiac surgery is becoming increasingly safer and mortality is a rare event. Now, much more attention is being placed on quality-of-life outcomes and morbidity following cardiac surgery rather than looking at mortality alone. Currently, neither EuroSCORE nor STS score include frailty assessment in their risk models; however, there is accumulating evidence highlighting the importance of frailty as a predictor for perioperative morbidity, and perhaps mortality. The challenge with including frailty in these risk scores is that there are a range of tools for assessing frailty and many of them have subjective elements, introducing the possibility of bias and a lack of reproducibility. Risk-scoring models work best when there are objective measures. The results of the studies reported in this review suggest that AGEs may offer that reliable and objective measure of frailty that is desired. The correlation between AGEs and perioperative morbidity and mortality suggests that they may be a useful measure to be included in future versions of risk-scoring models in cardiac surgery—offering an opportunity to recognise the importance of frailty which is currently not considered in the current models. Whilst EuroSCORE focuses on mortality risk, there are STS morbidity scores and it would seem that frailty assessment would particularly fit as an adjunct to the current variables considered since there appears to be a particular association with frailty, and AGEs, and the incidence of perioperative complications.

The study by Hoffman et al. explored the use of AGEs as a predictor in comparison to EuroSCORE and the STS score and further provides evidence for using AGEs as an adjunct variable. They demonstrated that skin autofluorescence (sAF) was a better predictor of mortality and morbidity in comparison to the STS score and EuroSCORE II [32]. It is, therefore, likely that the best scenario will be to include a measure of frailty in these risk-scoring systems to increase their ability to predict morbidity and mortality, with AGEs being a good contender as an objective measure of frailty.

### 5.1. Why Might AGEs Be Associated with Post-Operative Morbidity and Mortality?

Given the observation that there is association with AGEs and post-operative outcomes, the question that arises is why this should be. There have been several proposed mechanisms by which AGEs may increase the risk of post-operative complications that have been identified. There appears to be common mechanisms by which higher levels of AGEs are thought to cause a range of post-operative complications including activation of inflammatory mediators, inducing cell cycle arrest, causing endothelial dysfunction, and by promoting oxidative stress.

#### 5.1.1. Post-Operative Complication of Cardiovascular Disease

Several studies have revealed an association between AGEs and cardiovascular complications. These increased cardiovascular complications may be explained through a variety of mechanisms, including the ability of AGEs to cross-link respiratory chain mitochondrial proteins to enhance ROS formation [58]. These ROS can oxidise low-density lipoprotein, or LDL, to promote atherosclerosis [59] but can also lead to endothelial dysfunction that can cause vasoconstriction and hypertension [60]. Additionally, AGEs can cross link to collagen and elastin that increases arterial and myocardial stiffness [61] and reduces collateral vessel development through impaired angiogenesis [62].

#### 5.1.2. Post-Operative Complication of Infections

In terms of the increased risk of infections post-operatively, this may not only be explained by the fact that AGEs are responsible for overactivation of pro-inflammatory pathways [63], which disrupts the body’s normal immune response, making the body more susceptible to infections, but also by the fact that studies have shown AGEs to facilitate viral cell entry by activating proteins like CD147 too [64].

#### 5.1.3. Post-Operative Complication of Kidney Disease

Higher AGEs may lead to post-operative complications related to kidney disease because AGEs can cause overproduction of TGF-b1 (an inflammatory cytokine) in glomeruli [65] and increase levels of ROS through the activation of NADPH oxidase [66], inducing podocyte cell cycle arrest [67]. This can cause damage to the cellular components of the kidney, leading to fibrosis and kidney failure.

#### 5.1.4. Post-Operative Complication of Transplant Graft Failure

Studies have shown that AGEs are able to activate macrophages and monocytes that then release inflammatory mediators leading to tissue damage and complications like increased transplantation dysfunction [68]. Furthermore, higher AGEs may accelerate the progression of arteriosclerosis too; for example, the transplanted organ [69] which has been associated with graft failures [70].

### 5.2. How Should AGEs Be Measured?

The data above provides compelling evidence for an association between AGEs and perioperative morbidity and mortality and provides an explanation of why. The question which follows is, then, how should we use this knowledge in clinical practice? The first thing to consider is that the studies have used two different methods of measuring AGEs—skin autofluorescence and ELISA measurement of RAGEs. At present there appears to be no consensus on the most effective way of measuring AGEs and there is, therefore, no standardised method to measure them [71]. However, there are clear advantages and disadvantages of the two main methods. Using ELISA is more invasive as it requires a blood test and has issues with interference with glycation free adducts, i.e., glycated amino acids [71]. However, using sAF poses several limitations as well. Firstly, the fluorescence of AGEs can be affected by absorption by melanin, thus falsely reducing the value of AGEs in people with higher melanin concentrations in their skin [72]. Furthermore, studies have suggested that sAF can be affected by the application of skin creams and soaps [73]. If AGEs are to become incorporated as a standardised frailty assessment tool, there will need to be some clarity on the most accurate and reliable assessment method for measuring them. We propose that this could potentially be through prospective studies where AGEs are measured using both sAF and ELISA and then the occurrence of post-operative complications are monitored for a defined time period. The statistical association between post-operative complications and sAF, compared to post-operative complications and AGEs measured through ELISA, should then be compared. Additionally, to work out the most optimal time to measure AGEs pre-operatively, prospective studies can be performed by measuring the AGEs at different time frames pre-operatively and then establishing which of these times has the greatest association to post-operative outcomes.

### 5.3. Can Anything Be Conducted for Patients with High AGEs Levels?

The logical question which arises from the data reported here is the following: can we do anything to lower the risk of morbidity and mortality in patients with high AGEs levels? Whilst having a tool to assess risk of morbidity is nice, what would make it even more useful, clinically, is if there was something that could be performed to help patients found to have high AGEs levels to reduce their risk of post-operative complications. One can foresee the utility of AGEs measurement as a tool to identify patients, for whom some intervention should be offered preoperatively, to optimise them with the aim of reducing their subsequent risk from undergoing surgery. So, can anything be conducted? The answer appears to be ‘yes’.

Animal studies have shown that restricting calories or the diet of rats reduced the rate of accumulation of AGEs in skin collagen in comparison to their counterparts who had unrestricted diets [74]. Other human studies show that eating diets high in Maillard reaction products (the precursor molecules of AGEs) leads to higher plasma carboxymethylysine (a specific AGE) [75]. Diets high in Maillard reaction products are those where food preparation involves heating, e.g., fried, boiled, and toasted foods [76]. There are also studies suggesting that consuming high levels of fructose, compared to other types of sugars, may be associated with higher levels of accumulation of AGEs [77]. So, these studies suggest that modifying patients’ diets can positively impact AGEs level. Some studies have shown that even just changing the cooking methods, whilst using the same ingredients, can significantly change the level of AGEs after 2 weeks [78].

In addition to dietary changes, AGEs may also be reduced by other lifestyle changes like exercise. Studies have shown that obese rats that underwent regular exercise had lower levels of renal AGEs [79]. There is also evidence to suggest that exercise in humans can reduce AGEs level too [80] and this, therefore, may provide evidence for why prehabilitation before surgery can be very useful.

At present, there is limits to the literature on the impact that lowering AGEs has on the overall health of the patient and chronic diseases. But the evidence in this review suggests that this would offer a useful next step to assess the true utility of AGEs as a frailty measure.

### 5.4. Limitations

Our review is unfortunately subject to limitations. The first limitation is that the search strategy was restricted to only incorporate studies in the English language, potentially excluding valuable studies in other languages. A further limitation of this review is that it is likely subject to publication bias, as it is possible that studies that show a minimal or negligible association between AGEs and post-operative outcomes may not have published their results. Additionally, several studies that were included have used sAF as the measure of AGEs which, as discussed above, has some potential limitations in non-Caucasian populations [16]. That said, there are studies that have shown that adjustments can be made to algorithms that calculate AGEs based on sAF to increase their validity on different skin tones [81]. Future research and studies should consider exploring this and implementing changes to ensure the validity of sAF on a variety of skin tones, so that sAF can reliably be used to assess AGEs in patients from all ethnicities.

### 5.5. Future Directions

There are several interesting avenues of research to further explore. Firstly, there is strong evidence to suggest that AGEs are associated with both post-operative complications and frailty [18], yet there is a lack of research pertaining to the intersection or the causal relationships between all three areas. Also, it appears that measuring AGEs at different times in relation to the time of the surgery may change their ability and value as prognostic indicators [31,41]. This must be further explored to establish the most optimal time of measuring AGEs to extract their highest possible prognostic value. Additionally, the prognostic value of peri-operative increases in AGEs to predict patients at greatest risk of post-operative complications [35] must also be explored. Finally, there appears to be a relationship between AGEs and CRP levels [35]. This could potentially mean that CRP levels could also be used as prognostic biomarkers, which is particularly useful given that CRP is commonly measured in clinical settings.

## 6. Conclusions

The concept and importance of recognising frailty is becoming increasingly apparent in cardiac, and other, surgical practice. Since the risk of cardiac surgery has greatly reduced over time, it is becoming more important to evaluate morbidity and quality of life measures, rather than just mortality alone, when considering patients for surgery. This review provides evidence to suggest that AGEs offer a reliable and reproducible objective measure of frailty. Furthermore, there is an association between AGEs and perioperative complications. AGEs measurement preoperatively may, therefore, offer an opportunity to identify patients who have frailty, and the offer the possibility of intervention, such as prehabilitation, employed with the aim of reducing the risk of them experiencing complications after undergoing surgery. An objective measure of frailty may also offer an opportunity for inclusion in risk-scoring systems such as EuroSCORE and the STS score.

## Figures and Tables

**Table 1:** Summary of studies showing an association between frailty and AGEs.

Article	Month/Year	Type of Study	Method	Results
Drenth et al. [16]	October 2018	Observational cross-sectional study	5624 participants aged 65 years and older were taken from the LifeLines Cohort Study [17]. Linear regression conducted between sAF, number of physically active days, and physical functioning.	sAF and physical functioning was significantly associated, even when confounding variables considered.
Yabuuchi et al. [18]	October 2020	Observational cross-sectional study	37 patients undergoing maintenance dialysis at Juntendo University hospital in Japan. Serum AGE measured and frailty assessed using timed up and go test.	Serum AGE was significantly associated with increased frailty status. Slowness and weight loss were the variables with greatest levels of association with higher AGE levels.
Waqas et al. [19]	October 2022	Prospective cohort study	2521 participants were taken from the Rotterdam study [20] and AGEs measured as sAF using AGE reader. Frailty was measured using both Fried and Rockwood’s criteria. Multivariate regression was carried out, adjusting for confounders like age, renal function, smoking, and socioeconomic status.	sAF was associated with both pre-frailty and a higher frailty index.
Butcher et al. [21]	April 2024	Prospective cohort study	391 diabetic patients from European cohorts enrolled in the FRAILOMIC project [22], followed over 6 years. Patients were divided into frail and non-frail patients with frailty assessed using Fried’s criteria. Serum sRAGE measured using ELISA.	Stronger association between sRAGE and mortality in those that were frail. Higher sRAGE was associated with higher mortality in both groups.
Iida et al. [23]	June 2024	Observational cross-sectional study	AGE was measured using sAF. 264 participants, from the 2022 Yakumo Study [24], were divided into two groups according to presence or absence of fall risk and factors associated with fall risk were investigated.	Fall risk group had a higher age, sAF, and a higher proportion of locomotive syndrome. sAF was independently associated with fall risk in older adults, even after factoring in confounders.
Kuiper et al. [25]	November 2024	Prospective cohort study	2382 participants from the Doetinchem Cohort Study [26]—measured sAF, MetaboHealth, frailty index, and frailty phenotype using Fried’s criteria. Confounding variables considered were as follows: age, sex, socioeconomic status, and season.	sAF was associated with higher frailty index scores. Associations between sAF and frailty and pre-frailty were still seen when data was adjusted for confounders.
Takei et al. [27]	December 2024	Observational cross-sectional study	559 participants visiting Ehime University Hospital in Japan for check-up. Dermal AGE accumulation was measured using sAF.	Risk of cognitive impairment is significantly associated with sAF, especially in men. There is a closer association between sAF and physical frailty in men than in women.

**Table 2:** Summary of studies exploring association of AGEs with post-operative outcomes.

Author	Year	Country of Study	Sample Size	Surgery Type	Measurement of AGE	Complications and Outcomes Measured	Results	Summary of Findings
**Cardiac Surgery**								
Simm et al. [30]	2007	Germany	75	Cardiac surgery—CABG	Using ELISA to measure levels of carboxymethyl lysine (an AGE) from pericardial fluid, taken after pericardial incision was made.	Ventilation time, ejection fraction, AF, low cardiac output, MI, respiratory insufficiency, pneumonia, ICU length of stay.	Statistical association between increasing AGE and age of patient (r = 0.379, *p* = 0.0008). Increased AGEs correlated with increased levels of cardiac complications (*p* = 0.018) but not pulmonary complications (*p* = 0.279).	Increased AGEs are statistically associated with increased cardiac complications (AF, low cardiac output, post-operative MI) but not with increased pulmonary complications (respiratory insufficiency, pneumonia and prolonged ventilation time).
Creagh-Brown et al. [31]	2013	London, UK	129	Cardiac surgery—CABG, valve replacement, or more than one valve being repaired/replaced.	sRAGE measured using ELISA before surgery and 2 h after surgery.	Duration of mechanical ventilation, acute lung injury presence, hospital length of stay, ICU length of stay, mortality.	Plasma levels of sRAGE were significantly higher in patients 2 h after surgery (*p* < 0.0001). Preoperative sRAGE levels appeared to be the strongest predictor of hospital length of stay and other outcomes (*p* < 0.001). Post-operative sRAGE did not have any such correlations.	Preoperative sRAGE is a strong predictor of cardiac surgical outcomes, but not post-operative sRAGE.
Hoffman et al. [32]	2020	Germany	758	Cardiac surgery—CABG or aortic valve replacement or both.	sAF was measured at time of preoperative patient visit.	Mortality, MI, new AF or VF, reintubation, prolonged ventilation > 24 h, dialysis post-operatively, Cr > 350 μmol/L, neurological deficit > 24 h, wound healing issue, reoperation.	sAF was independently associated with higher morbidity (*p* < 0.0001)—independent of age or lower LVEF. sAF could predict in-hospital mortality (*p* = 0.0003) with greater significance compared to other existing scores, e.g., EUROSCORE II (*p* = 0.12) and STS Score (*p* = 0.02).	sAF is an independent predictive marker of mortality and morbidity after cardiac surgery and is at least comparable to other predictive scoring systems, if not better.
Reichert et al. [33]	2022	Germany	95	Cardiac surgery—CABG.	Both sRAGE and sAF were measured pre-operatively.	Observed over a period of 3 years post-operatively to monitor early and late cardiovascular and cerebrovascular outcomes.	sAF and sRAGE serum levels were not significantly associated with a poorer cardiovascular outcome after CABG (*p* = 0.257).	Insufficient evidence to suggest that sAF and sRAGE were associated with poorer cardiovascular outcome after CABG.
Smoor et al. [34]	2023	2 centres in Netherlands	555	Cardiac surgery—open procedures.	sAF was measured preoperatively in participants aged over 70.	Disability, death, reoperation, reintubation, stroke, readmission to ICU, life-threatening bleeding, renal replacement therapy, respiratory insufficiency.	sAF was associated with dependent living (*p* < 0.006) and had worse physical health related quality of life (*p* = 0.0025). Higher sAF was also associated as a predictor of poor outcomes (when taking death or disability as a composite endpoint) 1 year after surgery (*p* < 0.001).	Higher sAF levels predict death and disability 1 year after cardiac surgery.
**General Surgery**								
Pol et al. [35]	2011	Netherlands	40	General surgery—elective colorectal surgery for malignancy.	sAF was measured day before operation and every day after until discharge.	Systemic infections, MI, pulmonary complications, anastomotic leakage.	There was an increase in sAF by 19 +/− 2% after surgery. Both increased preoperative sAF and perioperative increases in sAF were correlated with development of post-operative complications (*p* < 0.01). Changes in sAF correlated to changes with CRP (*p* = 0.03).	Both increased preoperative sAF and perioperative increases in sAF may be useful to predict post-operative complications.
Neto et al. [36]	2017	Germany, USA, Netherlands	242	General surgery—open abdominal surgery.	sRAGE measured directly after and 5 days after surgery.	Post-operative pulmonary complications measured up to 5 days after surgery, including ARDS, atelectasis, pleural effusion, pulmonary oedema, etc.	Median levels of sRAGE did not change after surgery (*p* = 0.783). sRAGE levels were not associated with post-operative pulmonary complications (*p* = 0.132).	sRAGE levels are not statistically associated with post-operative pulmonary complications and so insufficient evidence to suggest that they have prognostic capacity.
Choi et al. [37]	2019	Seol, South Korea	46	General surgery—laparoscopic colectomy.	sRAGE measured using ELISA 20 min after induction of anaesthesia, at skin closure and 24 h after operation.	Complications measured during first six post-operative days including the following: duration of recovery room stays, diet day, duration of hospital and ICU stay, and respiratory complications.	Intra- and post-operative sRAGE was significantly associated with development of post-operative respiratory complications (*p* < 0.00001).	Intra- and post-operative sRAGE can be used to predict development of post-operative respiratory complications.
Krasnodebski et al. [38]	2021	Warsaw, Poland	130	General surgery—liver resection for suspected malignancy.	AGEs were measured using sAF in the immediate preoperative period.	AKI within seven post-operative days was measured.	32 patients had an AKI with 9 having a severe AKI. sAF was independently associated with AKI development (*p* = 0.047) and sAF-predicted operative time (*p* = 0.046).	sAF is a significant predictor of AKI, even after confounding variables are factored in.
Morawski et al. [39]	2022	Warsaw, Poland	54	General surgery—subcostal laparotomy for suspected GI malignancy.	sAF was measured before and after surgery.	Presence or absence of incisional hernias were measured 1–2 years after surgery. BMI and diabetes was monitored too.	There was no difference in sAF between patients with and without incisional hernias when BMI and diabetes were individually factored in (*p* = 0.587 and *p* = 0.669, respectively).	Lack of evidence to suggest that sAF could be used to predict incisional hernias after surgery.
**Thoracic surgery**								
Calfee et al. [40]	2007	USA	20	Thoracic surgery—lung transplantation or heart lung transplantation.	sRAGE levels measured within mean of 4 h of cross clamp release via ELISA.	Bronchiolitis obliterans syndrome (BOS), mortality (up to 1 year later), duration of mechanical ventilation, ICU length of stay.	Doubling the sRAGE levels, increased mechanical ventilation time by 26 h and ICU stay by 1.76 days, when adjusted for ischemia time (*p* = 0.018). No association between sRAGE and mortality or presence of BOS at 1 year. Neither PGD score nor ischemia time predicted post-operative complications.	sRAGE levels at 4 h were significantly correlated with both duration of mechanical ventilation and ICU stay, and was a better indicator than primary graft dysfunction score.
Shah et al. [41]	2013	USA	106	Thoracic surgery—lung transplant.	sRAGE measured 6 h and 24 h after surgery.	Development of BOS.	Average time to develop BOS was 3.4 ± 1.8 years. sRAGE measured 6 and 24 h after surgery were associated with increased risk of BOS (*p* = 0.02 and *p* = 0.01, respectively).	sRAGE levels were significantly associated with bronchiolitis obliterans syndrome post-operatively.
Nakao et al. [42]	2022	Japan	152	Thoracic surgery—wedge resection, segmentectomy and lobectomy.	sRAGE levels measured using ELISA just before surgery and 1 month after surgery.	Post-operative acute exacerbation of interstitial lung disease was measured.	Post-operative AE-ILD developed in 17 patients. Lower sRAGE levels were significantly associated with development of post-operative AE-ILD, independently of confounding variables (*p* = 0.024).	Lower levels of sRAGE were associated with occurrence of post-operative AE-ILD, especially in lobectomy patients.

Abbreviations: AE-ILD = Acute Exacerbation of Interstitial Lung Disease; AF = Atrial Fibrillation; AGE = Advanced Glycation End products; AKI = Acute Kidney Injury; ARDS = Acute Respiratory Distress Syndrome; AU = Arbitrary Units; BMI = Body Mass Index; BOS = Bronchiolitis Obliterans Syndrome; CABG = Coronary Artery Bypass Graft surgery; Cr = Creatinine; ELISA = Enzyme-linked Immunosorbent Assay; EuroSCORE II = risk prediction model used in cardiac surgery; ICU = Intensive Care Unit; LVEF = Left Ventricular Ejection Fraction; MI = Myocardial infarction; PGD score = Primary Graft Dysfunction score; sAF = skin Auto-fluorescence; sRAGE = serum Receptor levels of Advanced Glycation End products; STS Score = Society of Thoracic Surgeons Score; TIA = Transient Ischaemic Attack; and VF = Ventricular Fibrillation.

**Table 3:** Table of Excluded Studies.

Study	Reasoning for Exclusion
Shohat et al. [43]	This article was not directly measuring AGEs through sAF or sRAGEs, but was measuring AGEs indirectly as it was measuring fructosamine which becomes degraded into AGEs.
Chen et al. [44]	This article was not directly measuring AGEs through sAF or sRAGEs, but was measuring AGEs indirectly as it was measuring glycated albumin which is a precursor of AGE but not an AGE itself.
Zhang et al. [45]	This article was not directly measuring AGEs through sAF or sRAGEs, but was measuring AGEs indirectly as it was analysing RIP3K, which is not an AGE but was shown to be interacting with sRAGE instead.
Lagier et al. [46]	Upon detailed full text analysis, it was apparent that this article was not looking at post-operative complications but was looking at how different types of ventilation affect sRAGE.
Redondo et al. [47]	After a discussion, a consensus was reached between the researchers that since the main intervention examined was coronary angioplasty, which is minimally invasive, this would not be considered as a surgical procedure and, therefore, was excluded.
Bohm et al. [48]	After a discussion, a consensus was reached between the researchers that since the main intervention examined was pulmonary vein isolation, which is minimally invasive, this would not be considered as a surgical procedure and, therefore, was excluded.
González-Ferrero et al. [49]	After a discussion, a consensus was reached between the researchers that since the main intervention examined was atrial fibrillation ablation, which is minimally invasive, this would not be considered as a surgical procedure, and therefore, was excluded.
Hartog et al. [50]	After a discussion, consensus was reached to exclude this since sAF was measured 73 months after surgery.
Kottmaier et al. [51]	After a discussion, a consensus was reached between the researchers that since the main intervention examined was atrial fibrillation ablation, which is minimally invasive, this would not be considered as a surgical procedure and, therefore, was excluded.
Perkins et al. [52]	Upon detailed full text analysis, this text was not analysing the relationship between sRAGE and acute lung injury, but was assessing the relationship between salmeterol treatment and acute lung injury.

## Supplementary Materials

The following supporting information can be downloaded at: https://www.mdpi.com/article/10.3390/jcm14176176/s1, File S1: PICO framework; File S2: Fully detailed search strategy; Table S1: Table of summary statistics explored in greater detail; Table S2: Table summarising the risk of bias assessment as per the ROBINS-framework [53]; Table S3: Assessment of certainty of evidence based on GRADE framework [54].

## Abbreviations

The following abbreviations are used in this manuscript:

AGEs	Advanced glycation end products
AKI	Advanced kidney injury
CRP	C-reactive protein
ELISA	Enzyme-linked immunosorbent assay
sAF	Skin autofluorescence
sRAGE	Skin Receptor of Advanced glycation end products
