# Supplementary material for: The Use of Advanced Glycation End-Product Measurements to Predict Post-Operative Complications After Cardiac Surgery
Source: J Clin Med. 2025 Sep 1;14(17):6176. doi: 10.3390/jcm14176176 (PMC12429286; doi:10.3390/jcm14176176)
Supplement: Supplementary file 1 [file jcm-14-06176-s001.zip › File S1 - PICO.pdf]

Supplementary Materials:

File S1: PICO (Population, Intervention, Comparison, Outcomes) Framework

Population: We included studies on adult patients (age $\geq$ 18 years) undergoing a surgical procedure.

Intervention: We considered all surgical procedures that involved making a major surgical incision or a laparoscopic procedure. Less invasive procedures e.g. coronary angioplasty and stenting were not included as the invasiveness of the procedure may affect the association between AGEs and post-operative complications. Examples of procedures that were not included include: pulmonary vein isolation (PVI), catheter ablation of atrial fibrillation, coronary angioplasty since these procedures were minimally invasive.

Outcomes: Our systematic review evaluated all types of post-operative complications and outcomes, including (but not limited to) mortality rate, cardiac and pulmonary complications, graft dysfunction (where applicable), ventilation time, reintubation, organ failures, etc. No post-operative complication nor time frame was specified in order for us to spot as many trends as we could within the data and to identify if certain post-operative complications had a greater association with AGEs.

Further exclusion criteria included excluding articles that were not written in the English language and excluding studies where the data was not obtainable or where the trial or study was not yet complete. Note that unpublished manuscripts and conference papers were deemed eligible for inclusion.
