# Supplementary material for: The Use of Advanced Glycation End-Product Measurements to Predict Post-Operative Complications After Cardiac Surgery
Source: J Clin Med. 2025 Sep 1;14(17):6176. doi: 10.3390/jcm14176176 (PMC12429286; doi:10.3390/jcm14176176)
Supplement: Supplementary file 1 [file jcm-14-06176-s001.zip › File S2 - Search Strategy.pdf]

File S2: Fully Detailed Search Strategy:

MEDLINE (through OVID):

1. ("advanced glycation end produc$" or "advanced glycation end-produc$").ti,ab.
2. "skin autofluorescen$".ti,ab.
3. (post-operat$ or "post operat$" or outcom$ or complicatio$ or predictor$).ti,ab.
4. Specialities, Surgical/
5. Glycation End Products, Advanced/
6. Postoperative Complications/
7. 1 or 2 or 5
8. 3 or 4 or 6
9. 7 and 8
10. Limit 9 to (english language and full text and humans and "all adults" and "remove preprint records")

Time frame from 1946 to 20th February 2025 since the database only had records starting from 1946

Embase:

1. ("advanced glycation end produc$" or "advanced glycation end-produc$").ti,ab.
2. "skin autofluorescen$".ti,ab.
3. (post-operat$ or "post operat$" or outcom$ or complicatio$ or predictor$).ti,ab.
4. Advanced glycation end product/
5. Postoperative complication/
6. 1 or 2 or 4
7. 3 or 5
8. 6 and 7
9. Limit 8 to (full text and human and english language and "remove medline records" and "remove preprint records" and (adult <18 to 64 years> or aged <65+ years>))

From start of records to 20th February 2025

Cochrane:

1. (advanced NEXT glycation NEXT end NEXT produc*).ti,ab,kw OR (advanced NEXT glycation NEXT end-produc*):ti,ab,kw OR (skin NEXT autofluorescen*).ti,ab,kw.
2. (post NEXT operat*):ti,ab,kw OR (post-operat*):ti,ab,kw OR (surgical outcom*):ti,ab,kw OR (surgical complication*):ti,ab,kw
3. MeSH descriptor: [Specialities, Surgical] explode all trees
4. MeSH descriptor: [Glycation End Products, Advanced explode all trees
5. MeSH descriptor: [Postoperative Complications] explode all trees
6. #1 OR #4
7. #2 OR #3 or #5
8. #6 AND #7

From start of records to 20th February 2025

Clinical Trials.gov:

("advanced glycation end products" OR "skin autofluorescence") AND ("post-operative" OR "post operative" OR "surgical complication" OR "surgical outcome")

From start of records to 20th February 2025

Scopus:

1. TITLE-ABS ("advanced glycation end produc?" OR "advanced glycation end-produc?" OR "skin autofluorescen?")
2. AND TITLE-ABS ("post-operat?" OR "post-operat?" OR "complicati?" OR "predictor?")
3. AND (LIMIT-TO (LANGUAGE , "English"))
4. AND (LIMIT-TO (EXACTKEYWORD , "Human") OR LIMIT-TO (EXACTKEYWORD , "Humans") OR LIMIT-TO (EXACTKEYWORD , "Adult"))

From start of records to 20th February 2025

Online Resources and Browsing (as per PRISMA-S guidelines):
Date conducted: 20th February 2025. Portal/ URL: https://scholar.google.com/. Search terms: ("advanced end glycation end product" OR "skin autofluorescence") AND ("surgical complications" OR "postoperative" OR "predictor NEAR/5 surgery"). Notes: All 24 pages that appeared were screened (n = 230 results)

Other Methods - Google Scholar:
As per the PRISMA-S (extension to PRISMA statement guidelines), we also conducted a Google Scholar related articles search on all studies and articles that had been selected from the above methods on the 6th of March 2025 to ensure that all possible studies linking the association between AGEs and post-operative complications could be found. This was especially important since AGEs is a relatively new concept in the field and therefore literature on the subject is limited, making it important to find all pre-existing literature.

Search Strategy Development Process:
6 known relevant studies were used to identify studies within databases. We began considering appropriate search terms by identifying words present in the titles and abstracts of these studies. An outline for a search strategy was created and used to find more studies, from which additional search terms were identified. The search strategy was initially developed by a medical student in our team, with the final strategy being peer reviewed by an experienced cardiothoracic surgeon within our team. In order to further check and validate our search strategy, the search strategy was tested to see if it could identify the original 6 known studies that were found, and all 6 were indeed found.
