# Supplementary material for: The Use of Advanced Glycation End-Product Measurements to Predict Post-Operative Complications After Cardiac Surgery
Source: J Clin Med. 2025 Sep 1;14(17):6176. doi: 10.3390/jcm14176176 (PMC12429286; doi:10.3390/jcm14176176)
Supplement: Supplementary file 1 [file jcm-14-06176-s001.zip › Table S.1 - Table of Summary Statistics In Greater Detail.pdf]

Table S.1: Table of summary statistics explored in greater detail

Study:	Exploration of Summary Statistics In Detail:
Simm et al. [30]	Statistical association between increasing AGE and age of patients (r=0.379, p = 0.0008)<br><br>Patients were then divided into three tertiles, according to their age. The patients in the highest age tertile were then analysed. Age of these patients in this group was 75.13 ± 0.63 years. The following conclusions were made of this 3rd tertile:<br><br>Increased AGEs also correlated with increased levels of cardiac and pulmonary complications.1 out of 26 patients in lowest tertile of AGEs had cardiac complications compared to 7 out of 25 patients in highest tertile of AGEs. 1 out of 26 patients in lowest tertile of AGEs had pulmonary complications compared to 3 out of 25 patients in highest tertile of AGEs.<br><br>Only cardiac complications showed statistical significance (p = 0.018) whilst pulmonary complications did not (p = 0.279).
Creagh Brown et al. [31]	Plasma levels of sRAGE were significantly higher in patients 2 hours after surgery than preoperatively (p < 0.0001). Preoperatively, plasma levels of sRAGE were 1.06 ng/mL, IQR 0.72–1.76, and post-operatively, plasma levels of sRAGE were 1.93 ng/mL, IQR 1.14–2.63.<br><br>Preoperative sRAGE is a strong statistically significant predictor of cardiac surgical outcomes but not postoperative sRAGE.<br>Hospital mortality was 5% out of a sample size of 129 patients. ICU length of stay median was 40.5 hrs (IQR of 21.7-49.9).<br><br>Preoperative sRAGE levels appeared to be the strongest predictor of hospital length of stay and other outcomes (p< 0.001). Median hospital length of stay was 8.9 days (IQR of 7.2-13.1).<br><br>Postoperative sRAGE did not have any such correlations
Hoffman et al. [32]	sAF (mean of 2.8 ± 0.6) was independently associated with higher morbidity (p < 0.0001) - independent of age, lower LVEF. A high morbidity outcome was defined by the study as 2 or more organ issues, this was seen in 118 of the 758 patients.

	Only sAF could predict in-hospital mortality (16 patients or 2.1% of sample size died) (p = 0.0003), whilst existing scores e.g. EUROSCORE II (mean of 2.27 ±2.01) (p = 0.12) and STS Score (mean of 1.67 ± 1.59) (p = 0.02) could not
Reichert et al. [33]	Median value for sAF was 2.7 au (IQR of 2.4-3.1) and sRAGE 568 pg/ ml<br>sAF and sRAGE serum levels were not significantly associated with a poorer cardiovascular outcome after CABG (p = 0.257) - risk ratio for sRAGE was 1.0 and for sAF was 1.401.
Smoor et al. [34]	sAF was associated with dependent living (p < 0.006) and had worse physical health related quality of life (p= 0.0025)<br>Median SAF level was 2.87 (IQR 2.40–3.20). In patients with high sAF levels, 10 out of 183 or 5.5% of patients were dependent in their daily living compared to 8 out of 372 (2.2%) patients with low sAF levels. 56 out of the 183 patients with high sAF levels (30.6%) had low physical health related quality of life, as opposed to 64 out of the 372 patients in the low sAF group (17.2%).<br><br>Higher sAF was also associated as being a predictor of poor outcomes (when taking death or disability as a composite endpoint) 1 year after surgery (p <0.001). Median sAF was 2.75 (IQR 2.40-3.20) in patients who either died or developed a disability as opposed to median value of 2.60 (IQR 2.23-2.97) in patients who did not have either of these two outcomes.
Pol et al. [35]	There was an increase of sAF by 19 +/- 2% after surgery. Both increased preoperative sAF and perioperative increases in sAF were correlated with development of postoperative complications.<br><br>Both increased preoperative skin AF (mean 2.15 ± 0 .6) and perioperative increases in sAF may be useful to predict post-operative complications (both p<0.01). Increase in perioperative sAF was 0.26+/- 0.05 in patients without complications compared to 0.57 +/- 0.1 in patients with complications.<br><br>Changes in sAF correlated to changes with CRP (r=0.39, p =0.03)
Neto et al. [36]	Median levels of sRAGE did not change after surgery (preoperatively median 1.9 µg/ml and IQR of 1.5 to 2.4 whereas the post-operatively median was 1.9 µg/ml and IQR of 1.5 to 2.5) (p =0.783)<br><br>Preoperative sRAGE levels were not associated with post-operative pulmonary complications (p = 0.132). In patients with pulmonary complications, preoperative sRAGE had a median value of 2.1 µg/ml and IQR

	of 1.7 to 2.5 whereas in patients without pulmonary complications, preoperative sRAGE had a median value of 1.8 µg/ml and IQR of 1.4 to 2.3.
Choi et al. [37]	Intra- and postoperative plasma sRAGE was useful to predict development of postoperative respiratory complications (defined as having 2 or more of the criteria mentioned) ($p < 0.00001$).
Krasnodebski et al. [38]	sAF was independently associated with AKI development ($p = 0.047$) and sAF predicted operative time ($p = 0.046$). Median sAF was 2.3 AU with IQR of 1.9-2.6 AU. Operative time median was 3.5 hours with IQR of 2.8-4.3 hours. 32 patients had an AKI with 6.9% or 9 having a severe AKI<br><br>Optimal cutoffs for sAF to prediction of AKI was 2.7 AU
Morawski et al. [39]	There was no difference in preoperative sAF between patients with and without incisional hernias when BMI and diabetes were individually factored in ($p = 0.587$ and $p = 0.669$ respectively)<br><br>Median preoperative sAF was 2.0AU (IQR of 1.8-2.5) in patients without incisional hernias and median preoperative sAF was 2.4AU (IQR of 2.1-3.0) in patients with incisional hernias
Calfee et al. [40]	Doubling the plasma RAGE levels, increased mechanical ventilation time by 26 hours and ICU stay by 1.76 days, when adjusted for ischemia time ($p = 0.018$). Median duration of mechanical ventilation was 18 hours, IQR was 12-31 hours. Median duration of ICU stay was 4 days, IQR of 3-7 days.<br><br>No association between serum RAGE and mortality or presence of BOS at 1 year. 3 patients or 20% of the survivors developed BOS. Neither PGD score nor ischemia time predicted postoperative complications
Shah et al. [41]	Average time to develop BOS was 3.4 ± 1.8 years<br><br>sRAGE measured 6 and 24 hours after surgery were associated with increased risk of BOS ($p = 0.02$ and $p = 0.01$ respectively). sRAGE measured at 6 hours in patients with BOS was 9112 ± 11405 pg/ ml compared to 6484 ± 6954 pg/ ml in patients without BOS. sRAGE measured at 24 hours in patients with BOS was 5249 ± 7213 pg/ ml compared to 2897 ± 2576 pg/ ml in patients without BOS.
Nakao et al. [42]	Postoperative AE-ILD developed in 17 patients<br><br>Lower sRAGE levels (< 547.4 pg/ ml) were significantly associated with development of postoperative AE-ILD, independently of confounding

	variables (p = 0.024). sRAGE levels. sRAGE levels were 719.9 ± 122.3 pg/mL in those with AE-ILD compared to 914.1 ± 45.6 pg/mL in those without AE-ILD.

Abbreviations: AE-ILD = Acute Exacerbation of Interstitial Lung Disease; AF = Atrial Fibrillation; AGE = Advanced Glycation End products; AKI = Acute Kidney Injury; ARDS = Acute Respiratory Distress Syndrome; AU = Arbitrary Units; BMI = Body Mass Index; BOS = Bronchiolitis Obliterans Syndrome; CABG = Coronary Artery Bypass Graft surgery; Cr = Creatinine; ELISA = Enzyme-linked Immunosorbent Assay; EuroSCORE II = risk prediction model used in cardiac surgery; ICU = Intensive Care Unit; IQR = interquartile range; LVEF = Left Ventricular Ejection Fraction; MI = Myocardial infarction; PGD score = Primary Graft Dysfunction score; sAF = skin Auto-fluorescence; sRAGE = serum Receptor levels of Advanced Glycation End products; STS Score = Society of Thoracic Surgeons Score; TIA = Transient Ischaemic Attack; VF = Ventricular Fibrillation
