# Supplementary material for: The Use of Advanced Glycation End-Product Measurements to Predict Post-Operative Complications After Cardiac Surgery
Source: J Clin Med. 2025 Sep 1;14(17):6176. doi: 10.3390/jcm14176176 (PMC12429286; doi:10.3390/jcm14176176)
Supplement: Supplementary file 1 [file jcm-14-06176-s001.zip › Table S.2- Table of Risk of Bias.pdf]

Table S.2: Table summarising the risk of bias assessment as per the ROBINS-framework [53]

Study:	Pre-Intervention Bias:		During Intervention Bias:	Post-Intervention Bias:				Overall Risk:	Explanation of Reasoning Behind Bias Assessment:
	Bias Due to Confounding Factors:	Bias in Selection of Participants in Study:	Bias In Classification of Intervals:	Bias Due to Deviations From Intended Interventions:	Bias Due to Missing Data:	Bias in Measurement of Outcomes:	Bias in Selection of Reported Result:		
Simm et al. [30]	Low risk	Low risk	Low risk	Low risk	Low risk	Low risk	Moderate risk	Moderate risk	Instead of finding trends and associations between all the participants they divided the data into tertiles for the biological age and tertiles for the AGE concentration as well and tried to find patterns within these tertiles rather than looking at associations within the entire patient sample. This was done because their 'data suggested a threshold for age as well as AGE concentration'

Creagh-Brown et al. [31]	Moderate risk	Moderate ri sk	Low risk	Low risk	Low risk	Low risk	Low risk	Modera te risk	There was a 'lack of standardisation of perioperative care' The study focused on selecting patients at higher risk 'with a higher EUROSCORE or who underwent more complex surgery'
Hoffman et al. [32]	Low risk	Moderate risk	Low risk	Low risk	Low risk	Moderate risk	Low risk	Modera te risk	Patients who had 'emergency operations, reoperations,' were excluded from the study and therefore their study population was a 'population with a low to medium risk profile' Mortality and morbidity were only measured between the 'time extending from cardiac surgery until the patient was discharged'
Reichert et al. [33]	Low risk	Serious risk	Low risk	Low risk	Moderat e risk	Low risk	Low risk	Serious risk	This study had an inclusion criteria involving only patients of 'Caucasian descent' to be used '7 patients dropped out' due to various reasons

Smoor et al. [34]	Moderate risk	Low risk	Low risk	Low risk	Moderat e risk	Low risk	Low risk	Modera te risk	‘In total 2.5% of the preoperative sAF data were missing’<br>Disability (one of the outcomes measured) was only recorded during follow-up and ‘disability at 12 months could be a reflection of preoperative disability’ and there were ‘significant baseline differences between patients with high and low SAF level that may have biased’ their results
Pol et al. [35]	Low risk	Moderate risk	Low risk	Low risk	Low risk	Low risk	Low risk	Modera te risk	‘Only elective surgery patients were included’ and ‘laparoscopic techniques were not used in the study’

Neto et al. [36]	Low risk	Moderate risk	Low risk	Low risk	Moderate risk	Moderate risk	Low risk	Moderate risk	Study recruited those who were 'with intermediate or high risk for post-operative pulmonary complications'<br>'900 patients underwent randomisation' but 'blood sampling was complete in 242 patients' only<br>They only 'sampled blood only directly after surgery and on post-operative day 5'
Choi et al. [37]	Low risk	Moderate risk	Low risk	Low risk	Low risk	Low risk	Low risk	Moderate risk	'None of the assigned patients were excluded from the study'<br>Lots of potential, different confounding variables were controlled for<br>'Single team of surgeons were used'<br>'Patients were 20-65 years and had an American Society of Anesthesiologists (ASA) physical status score of I to II'
Krasnodebski et al. [38]	Moderate risk	Moderate risk	Low risk	Low risk	Low risk	Low risk	Low risk	Moderate risk	'Patients with skin phototypes IV–VI in Fitzpatrick's classification, and those with body mass

									index (BMI) > 35 kg/m squared were excluded' 'AKI can occur due to multifactorial confounders that were not assessed like hypovolemia, sepsis, intravenous contrast administrations, and/or drug interactions'
Morawski et al. [39]	Moderate risk	Low risk	Low risk	Low risk	No information	Low risk	Low risk	Moderate risk	'Data on patient's BMI and diabetes were collected preoperatively' but other confounding variables were not considered
Calfee et al. [40]	Low risk	Low risk	Low risk	Low risk	Low risk	Moderate risk	Low risk	Moderate risk	Study only measured 'plasma data from first 4 hours after reperfusion' and 'could not differentiate between RAGE generated in allograft donor and that generated in allograft recipient'
Shah et al. [41]	Low risk	Moderate risk	Low risk	Low risk	Low risk	Moderate risk	Low risk	Moderate risk	This study 'excluded all early deaths' 'Plasma samples were obtained in citrated tubes 6 and 24 hours after

									reperfusion of the lung allograft’
Nakao et al. [42]	Low risk	Low risk	Low risk	Serious risk	Moderat e risk	Low risk	Low risk	Serious risk	‘Of the 76 patients who underwent lung resection during this period, 23 had no postoperative samples’ so data was missing<br><br>The study showed that there was a reduction in sRAGE after lung resection but it did not elucidate if this was because of the operation or if “it was caused by the reduced lung volume and whether it promoted the development of post-operative AE-ILD’
