# Supplementary material for: The Use of Advanced Glycation End-Product Measurements to Predict Post-Operative Complications After Cardiac Surgery
Source: J Clin Med. 2025 Sep 1;14(17):6176. doi: 10.3390/jcm14176176 (PMC12429286; doi:10.3390/jcm14176176)
Supplement: Supplementary file 1 [file jcm-14-06176-s001.zip › Table S.3 - GRADE Assessment of certainty.pdf]

Table S.3 Assessment of certainty of evidence using GRADE framework [54]

Study:	Certainty of the Evidence (GRADE framework):	Comments:
Simm et al. [30]	Very low certainty	Rated down one level for indirectness
Creagh-Brown et al. [31]	Moderate certainty	Rated up one level for large magnitude of effect
Hoffman et al. [32]	Moderate certainty	Rated up one level for large magnitude of effect
Reichert et al. [33]	Low certainty	
Smoor et al. [34]	Low certainty	
Pol et al. [35]	Low certainty	
Neto et al. [36]	Low certainty	
Choi et al. [37]	High certainty	Rated up one level for large magnitude of effect
Krasnodebski et al. [38]	Low certainty	
Morawski et al. [39]	Low certainty	
Calfee et al. [40]	Low certainty	
Shah et al. [41]	Low certainty	
Nakao et al. [42]	Low certainty	Rated down for risk of bias
